# Investigations of the Optical and Thermal Properties of the Pyrazoloquinoline Derivatives and Their Application for OLED Design

**DOI:** 10.3390/polym12112707

**Published:** 2020-11-16

**Authors:** Gabriela Lewińska, Karen Khachatryan, Krzysztof S. Danel, Zoriana Danel, Jerzy Sanetra, Konstanty W. Marszałek

**Affiliations:** 1 Department of Electronics, Faculty of Computer Science, Electronics and Telecommunications, AGH University of Science and Technology; 30-059 Krakow, Poland; marszale@agh.edu.pl; 2 Department of Chemistry, Faculty of Food Technology, University of Agriculture in Krakow, 30-149 Krakow, Poland; karen.khachatryan@urk.edu.pl (K.K.); rrdanelk@cyf-kr.edu.pl (K.S.D.); 3 Institute of Physics, Faculty of Materials Science and Physics, Cracow University of Technology, 30-035 Krakow, Poland; zusatenko@pk.edu.pl; 4 Institute of Physics, Faculty of Physics of Mathematics and Computer Science, Cracow University of Technology, 30-841 Cracow, Poland; jsanetra@agh.edu.pl

**Keywords:** OLED, pyrazoloquinoline, differential scanning calorimetry, thermogravimetry

## Abstract

In this study, the photo-optical properties of the series of new 1H-pyrazolo[3,4-b]quinoline derivatives were investigated. Pyrazoloquinoline studies were conducted to explain the electroluminescent effect in organic LEDs. Absorption and photoluminescence spectra for the materials under consideration were examined, and quantum chemical calculations were made. Differential scanning calorimetric and thermogravimetric measurements were carried out for the manufactured materials. The phase situation of the materials was determined, and glassy transitions were detected for three of the investigated materials. Degradation temperatures were obtained. Single-layer luminescent diodes based on the ITO/PEDOT:PSS/active layer/Al scheme were fabricated. Current–voltage and brightness–voltage characteristics of the diodes were determined, ignition voltage was calculated, and electroluminescence types were determined.

## 1. Introduction

Since the experiments of Tang et al. [1,2] on light emission from organic materials proved to be successful, research concerning optoelectronics has achieved a new impetus, and even today the rate of discovery of new materials is not slowing. Organic light-emitting diodes (OLEDs) [3,4,5], a type of light-emitting diode, are produced from organic compounds. OLEDs [6,7,8], as a surface light source [9,10], are commonly used to build flexible displays [11,12,13], TV sets [14,15], memory devices [16,17], solar cells [18,19,20,21], or other portable devices [22,23,24]. These materials have several advantages: they can be easily modified [25], they are cheaper to produce than inorganic materials, and they are also more energy efficient [26,27,28,29].

Quinoline derivatives are well-known materials for optoelectronic applications [30,31,32]. Organic compounds that are used for this purpose must be characterized by a good efficiency of converting electricity into light, and the required spectra of electroluminescence [33,34]. In addition, these materials can be easily processed under the influence of temperature, electric charge flow, and light.

Quinoline is an organic compound with high electroluminescence efficiency [35] that is also easy to modify [36,37]. By modifying quinoline, it is possible not only to change the emission spectrum [38,39], but also other physical properties such as temperature sensitivity, resistance and photochemical changes or conductivity, and spatial properties [40,41,42]. A purposeful synthesis is important in order to obtain functional materials as a result [43,44]. The challenge is also to create a copolymer with a monomer which has better conduction properties and greater transmission of energy and electric charge [45].

Quinoline derivatives are well-known materials for optoelectronic applications [46], and can be modified relatively easily by changing the bases [47]. We present new derivatives of pyrazoloquinolines (PQs) and their optical properties, thermal tests [48,49], and manufactured devices in this paper.

## 2. Materials and Methods

Many modern pharmaceuticals, agrochemicals, and other advanced materials contain at least one fluorine atom, which usually has a specific function [50]. Because of this, fluorine is also used in the presented materials as one of the substituents. All materials were purchased from Sigma-Aldrich (Poznań, Poland).

**Figure 1:**
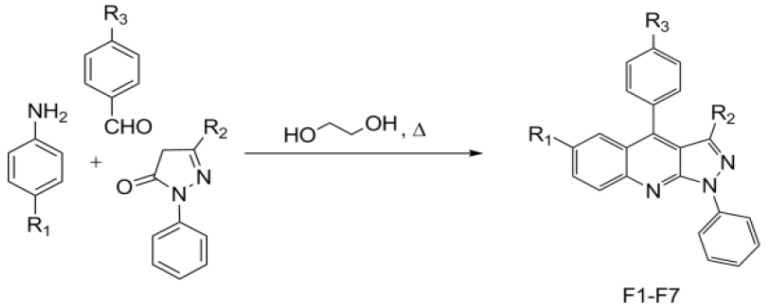
The one-pot procedure to prepare pyrazolo[3,4-b]quinolines F1–F7.

Equimolar amounts of aniline, aldehyde, and pyrazolone were heated to reflux in ethylene glycol for several hours. After cooling, methanol was added, and the mixture was heated to reflux for 30 min (Figure 1). Afterwards, yellow powders were collected and recrystallized from the appropriate solvents to deliver pure compounds. All compounds presented in Table 1, with codes F1 to F7, were obtained in the same manner.

## 3. Results

### 3.1. Quantum Chemical Calculations

The optimized ground-state geometries of compounds F1–F7 were estimated using the DFT model, employing hybrid functional Becke3–Lee–Yang–Parr (B3LYP) with a basis set B3LYP/6-31G(d,p) and B3LYP/6-31+G(d,p), to calculate the highest occupied molecular orbital (HOMO) and the lowest unoccupied molecular orbital (LUMO) values [51]. The differences in energy levels give a value of 0.3 eV, as calculated by both methods (see Table 2). The electron-donating moieties at the C-6 of the PQ skeleton raise the HOMO levels whenever the electron-withdrawing moieties decrease them. Similar trends are observed in LUMO energy levels. The calculations performed at B3LYP/6-31G(d) in chloroform give results lower by a factor of ~0.15 eV, regardless of HOMO/LUMO levels. In the following, we compare the measured optical absorption spectra with the excitation spectra, evaluated by the DFT/TDDFT (density-functional theory/time-dependent density-functional theory) method at the B3LYP/6-31G(d) level of theory.

The results of the TDDFT calculation of the highest maximum in λ_max_ superviolet in chloroform, and the oscillator strength (which is a measure of absorption intensity of new compounds) are presented in Table 3.

The results in Table 3 were obtained by inserting the optimized structure of the above into the TDDFT calculation method and calculating the wavelength (λ_max_) of the maximum of the absorption spectrum for each compound placed in chloroform. Simulated data are similar to the experimental results presented in Table 4.

### 3.2. Optical Properties

**Figure 2:**
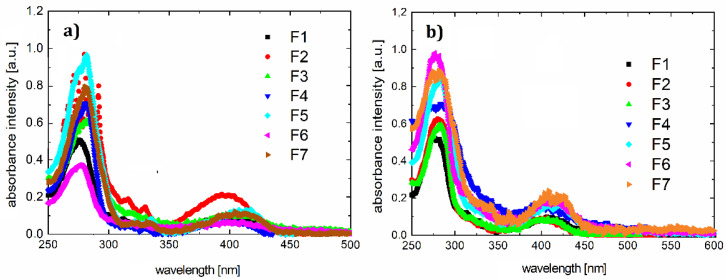
Absorption spectra for F1–F7 samples in chloroform (**a**) and thin film on quartz (**b**).

**Figure 3:**
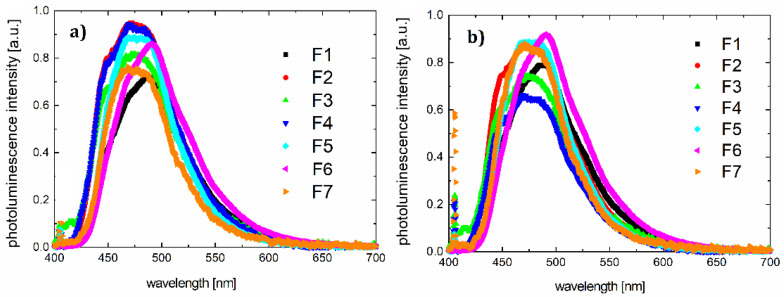
Photoluminescence spectra for samples F1–F7 in (**a**) chloroform and (**b**) thin film on quartz (λ_ex_ = 405 nm).

Absorption and photoluminescence spectra of all compounds were measured using the HR4000CG-UVNIR spectrometer by Ocean Optics. The graphs are presented in Figure 2 and Figure 3, respectively, and illustrate the quantitative dependence of absorbance versus wavelengths for the given organic materials.

The absorption of the pyrazoloquinoline derivatives was measured in a liquid state (solution in chloroform of spectroscopic grade that was purchased by Merck KGaA, Darmstadt, Germany), and in solid-state as a thin layer on quartz.

In the range of 250–650 nm, the optical absorption spectra of dyes are quite similar. The stronger absorption band appears in the spectral range of 240–340 nm, which can be associated with π* transition. The second absorption band is observed in the spectral range 415–650 nm and can be associated with transition n^*^. Maxima for individual bands and maximum photoluminescence are listed in Table 4.

Energy gaps obtained from theoretical calculations (presented in Table 2) are about 3.5 eV for all compounds F1–F7. This is confirmed experimentally by the results of the absorption measurments and indicates that the absorption spectra are almost identical, although HOMO and LUMO energy levels are different. Other energy levels should affect the ignition voltage and possibly the intensity of the light.

Subtle differences in peaks are caused by differences in the molecules. This may also be demonstrated by discrepancies in the Stokes shift values (differing by approximately 25 nm).

On the basis of the results presented, it can be concluded that the spectra of the individual compounds F1–F7 do not differ greatly (Figure 2a). In addition, the absorption spectrum in the solid state is slightly shifted towards long (about 10 nm) wavelengths, compared to those made in chloroform solution.

**Figure 4:**
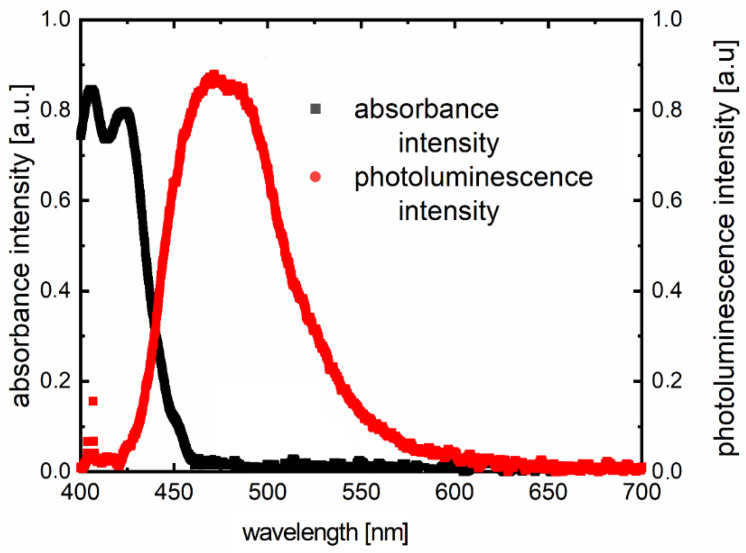
Example of the Stokes shift for sample F3 from Table 2. The photoluminescence has been excited by a diode laser (λ_ex_ = 405 nm).

Inserting substitutes does not affect the absorption spectrum significantly. The entire absorption is dominated by the skeleton of the molecule. The same luminescence spectrum behavior is observed. According to the absorption and photoluminescence spectra, Stokes shifts were determined as shown in Table 4. A sample chart for F3 is shown in Figure 4.

### 3.3. Thermogravimetry (TG) and Differential Scanning Calorimetry (DSC) Study

The thermogravimetric analysis (TGA), coupled with simultaneous differential thermal analysis (SDTA) and mass spectrometry evolved gas analysis (MS-EGA), was performed in a Mettler-Toledo 851e Thermo-Analyser Columbus, OH, USA) using 150 μL corundum crucibles under a flow of argon (80 mL/min), within the temperature range 30–600 °C with a heating rate of 5 °C/min. The simultaneous MS-EGA was performed in an online joined quadrupole mass spectrometer (QMS) (Thermostar—Balzers, Mettler-Toledo, Columbus, OH, USA).

**Figure 5:**
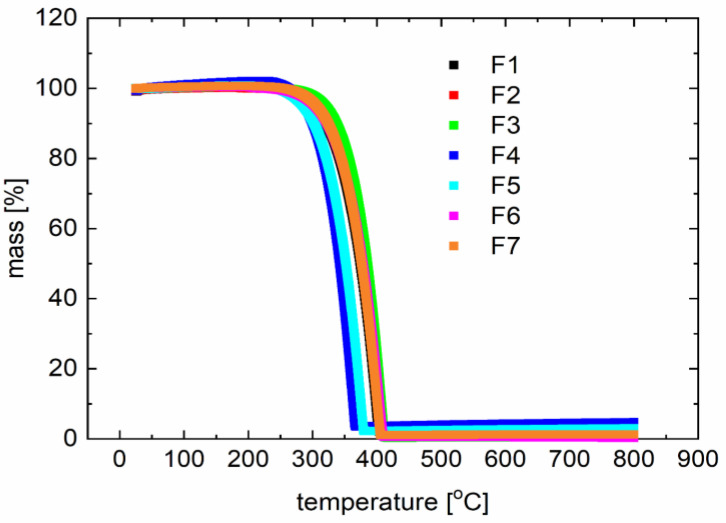
Typical thermogravimetric (TG) curves for derivatives F1 to F7 (described in Table 1).

Representative thermogravimetric curves of percent mass losses as a function of temperature for the investigated compounds are presented in Figure 5. The compounds have a similar degradation temperature for the materials shown in Table 5.

The degradation temperature is above 300 °C, which also confirms the broad application potential. The temperatures observed with the capillary method slightly deviate from those observed with differential scanning calorimetry measurements.

**Figure 6:**
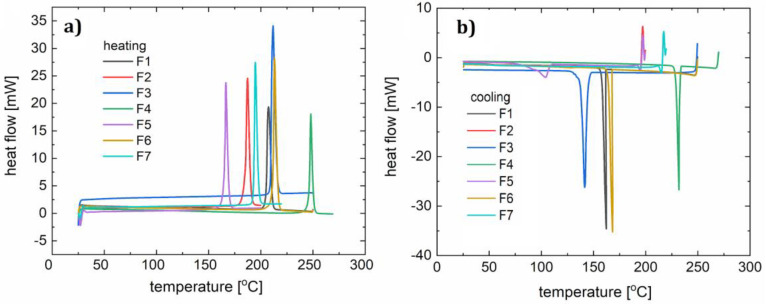
DSC curves for samples F1–F7: heating (**a**) and cooling (**b**).

Calorimetric measurements were performed using a Mettler-Toledo 821e calorimeter equipped with a Haake intracooler under an argon flow (80 mL/min) within a temperature range of 25–600 °C. The measurements were done in 40 μL aluminum crucibles closed by a lid with a hole (1 mm diameter) and 5–10 mg of sample. The heating and cooling curves are shown in Figure 6.

The melting range of all the synthesized compounds spans from 160 °C to nearly 210 °C (Table 5) in *p*-fluorophenyl series R_1_ = Et, *t*-Bu, Me, and OMe (F2–F5 and F7). The biggest contribution to increasing the melting point was the isopropyl moiety R_3_ = *i*-Pr. Exchanging the positions of a fluorine atom and *t*-butyl is also advantageous in enhancing thermal properties. Similar trends were previously observed, and those derivatives are highly crystalline compounds that decompose at around 400 °C.

**Figure 7:**
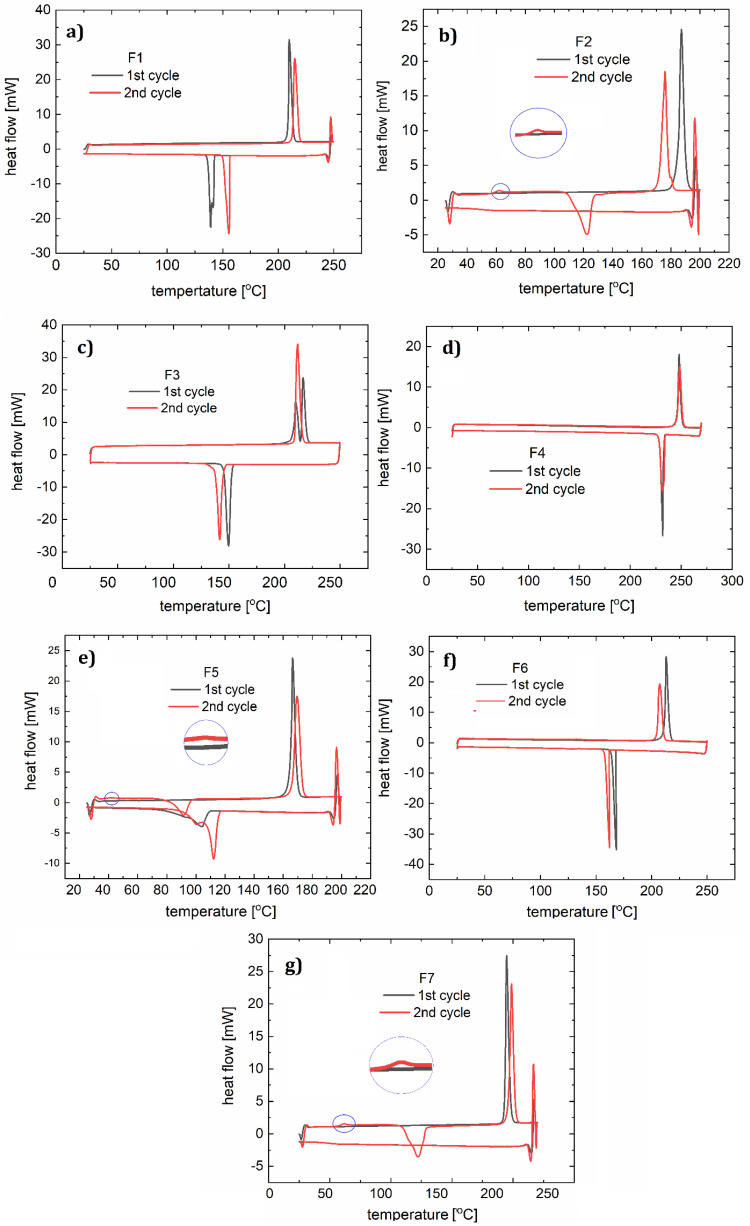
DSC curves with two heating and cooling cycles for: F1 (**a**), F2 (**b**), F3 (**c**), F4 (**d**), F5 (**e**), F6 (**f**), and F7 (**g**).

Two heating and cooling cycles were carried out for each material (Figure 7). For the polymer F1, during the first heating cycle (10 °C/min) the melting process (with a corresponding peak maximum at 210 °C) and a two-stage crystallization (peaks at 141 °C, 138 °C) occurred. Two peaks observed on the curve indicate an additional state of order of the molecules. In the second cycle (15 °C/min) the melting process (peak at 214 °C) and a crystallization (peak at 156 °C) were noted. For compound F2 in the first cycle (12 °C/min), a clear melting point was observed at 187 °C, followed by the creation of a glassy phase later during the process. During heating in the second cycle (10 °C/min), a softening glass transition at 62 °C was observed, followed by an endothermic process with a maximum of 123 °C (associated with the spontaneous crystallization of the cooled liquid) and double melting (at 175 °C and 180 °C). The lower temperature transition is responsible for the transition from the solid phase to another solid phase with more molecular disorder. In both the first and second cooling cycles (15 °C/min), a one-phase transition (at 150 °C and 114 °C) can be distinguished on the DSC curve of compound F3. During heating in the first cycle, we observed two phase transitions (peak maxima at 209 °C and 216 °C), and in the second cycle the DSC curve shows one maximum (212 °C). In this case, we also assume the creation of an additional metastate between the solid and liquid phases. For sample F4, both heating and cooling processes (10 °C/min and 15 °C/min) show one phase transition during heating (peak at 248 °C in the first cycle, and at 240 °C in the second cycle) and cooling (at 232 °C in the first cycle, and 230 °C in the second cycle).

The first heating cycle (10 °C/min) for compound F5 showed melting at 166 °C. During the cooling, a wide peak (104 °C) was observed. The second cycle (12 °C/min) showed glassy phase transitions (glass-liquid transition) with the maximum at 42 °C, and a following endothermic reaction at 91 °C, and melting at 170 °C. Since the glassy transition appeared, it can be concluded that this phase transition (corresponding with a peak at 104 °C) was not a complete crystallization with ordering of the molecules. During the second cooling we observed one wide peak with a minimum at 111 °C.

F6 in both heating and cooling cycles showed one phase transition for each condition (for heating 213 and 207 °C, and for cooling 168 and 161 °C). This behavior is similar to that of F4. For F7, a melting point at 192 °C was observed during heating. The second heating, like the first one, showed a glass transition at 61 °C, an endothermic process with the minimum at 61 °C and a melting point at 199 °C. The phase condition of F7 is therefore analogous to that of F2 and F5, according to the fact that small molecules are susceptible to spontaneous crystallization during OLED operation. Glassy transitions, as well as all the morphological changes in the operating temperature range of diodes, are undesirable and so it can be concluded that these compounds are not suitable for optoelectronic applications. Therefore, glass transitions exclude compounds F2, F3, and F5 from being used as single layers in OLED devices. However, doping into an appropriate host material was considered.

### 3.4. OLEDs

After the photophysical properties were demonstrated, single-layer electroluminescent diodes were made. The diodes were built based on the ITO/active layer/Al scheme, with indium tin oxide [52] as a transparent electrode (provided by Merck KGaA, Darmstadt, Germany) and evaporation aluminum as a second electrode.

The active layer in this simple, sandwich-like architecture was poly(*N*-vinylcarbazole) (PVK) matrix and dyes (the ratio of dye:PVK was about 1:100).

The light emission mechanism of an organic material involves the injection of a charge from the electrode to the material. For light-emitting devices based on organic compounds, we are almost exclusively concerned with recombination electroluminescence. Based on the kinetic equations describing the charge carrier population, we consider the injection-controlled and volume-controlled electroluminescence models [53].

**Figure 8:**
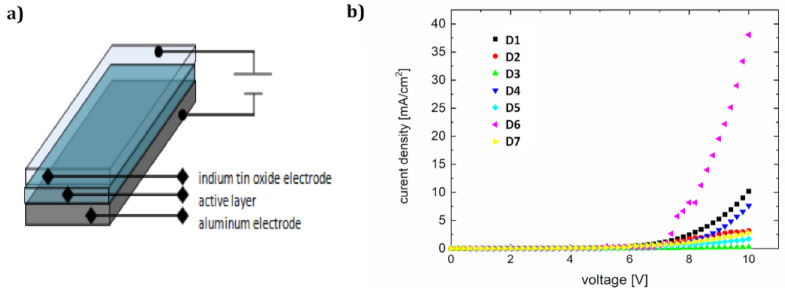
Device architecture (**a**) and current-voltage characteristics of diodes D1–D7 (**b**)**.**

**Figure 9:**
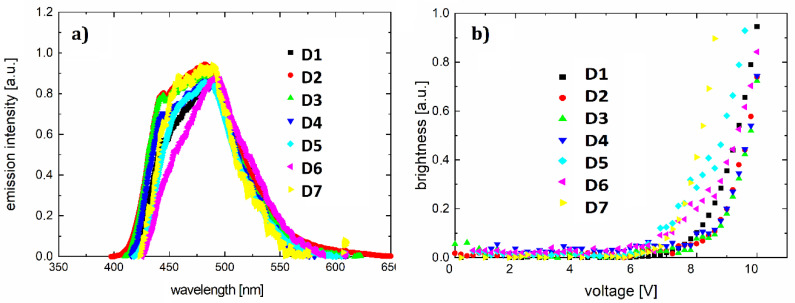
Electroluminescence spectra for D1–D7 (**a**) and brightness–voltage characteristics for diodes D1–D7 (**b**).

For ITO/PVK+F/Al diodes (shown in Figure 8a), designated as D1–D7, we obtained the current-voltage (Figure 8b) and brightness–voltage characteristics (Figure 9b). The lowest ignition voltage was for diodes D5 and D6 (6.2 and 6.4 V, respectively). A value of the ignition voltage of about 7 V was shown by devices D7, D1, and D4. OLEDs D2 and D3 showed an ignition voltage of about 8 V.

The resulting light-emitting spectra (Figure 9a) show a spectrum range from 425 to 570 nm, with full width at half maximum of about 100 nm. The electroluminescence maximum ranges from 481 nm (greenish-blue color) to 493 nm (bluish-green color). The parameters of the diodes are collected in Table 6.

## 4. Discussion

The electroluminescence flux model for injecting charge carriers from electrodes into the active layer of a case of thermal injection, above the barrier of the potential in the presence of a field, is defined by Equation (1):

(1) $$\Phi_{el}\propto {\left(j^{e},j^{h}\right)}^{n}$$

where $\;j^{h}\;\mathrm{and}\;j^{e}$ are hole and electron current density, and *n* is the current-electroluminescence coefficient. Depending on the complexity of the model, the flux can be described with more parameters.

**Figure 10:**
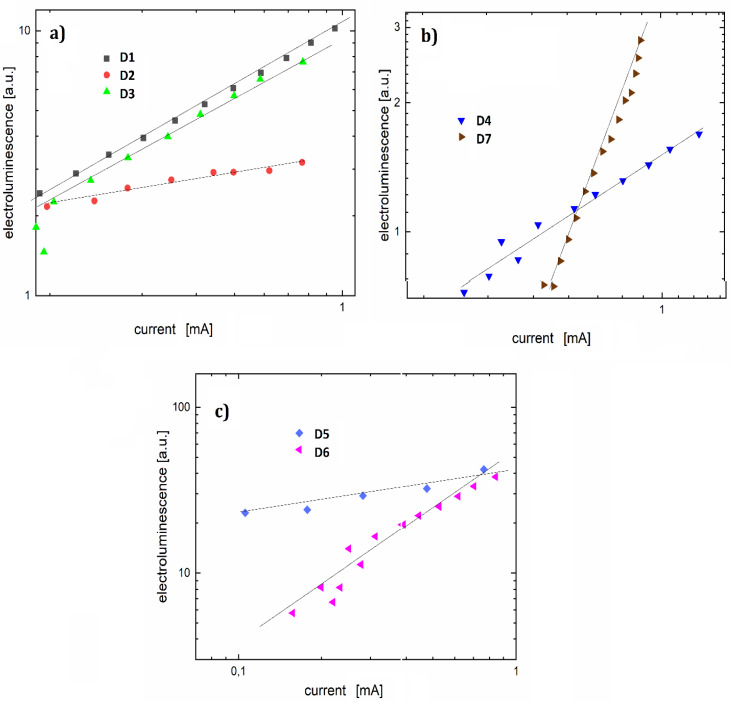
Log-log electroluminescence-current characteristics for organic light-emitting diodes (OLEDs) (**a**) D1, D2 and D3, (**b**) D4 and D7, (**c**) D5 and D6.

To determine the character of electroluminescence, the electroluminescence-current characteristics were presented on a double logarithmic scale for the investigated diodes (Figure 10). The linear function was adjusted to the characteristics. Based on the slope of the adjusted linear function, the current-electroluminescence exponent was determined. This allowed conclusions to be drawn about the electroluminescence model as well as the mechanism of charge-carrier injection.

Diode D1 with a coefficient *n* = 1.8 showed a reduction in the potential barrier for holes, comparable to the electron potential barrier. The value of the exponent for diode D5 is close to unity, which is characteristic of the volumetric electroluminescent model. However, it can also indicate strong electron scattering. For diodes D4 and D6, the slope factor *n* of 1.2 could be the result of electron trapping. Other devices have the following exponents: D2 = 4.2, D3 = 3.4, and D7 = 2.4. Analyzing the performance of individual luminescence devices can lead to the conclusion that the substituents do not affect the electroluminescence maximum. However, they definitely affect the ignition voltage and the mechanisms of electroluminescence.

To improve the device parameters, extra layers supporting the transport of holes or electrons are often used [54]. Diodes with an additional layer of poly(3,4-ethylenedioxythiophene)-poly(styrenesulfonate) (PEDOT:PSS) were pre-tested. Diodes of ITO/PEDOT:PSS/active layer/Al architecture were made and tested. Unfortunately, the ignition voltage increased, which is not favorable for diodes. The presented architecture (ITO/active layer/Al) is the best that was achieved.

## 5. Conclusions

This paper presents the synthesis of pyrazoloquinoline derivatives. HOMO and LUMO levels, as well as theoretical values of maximum absorption, were determined using quantum chemical simulations for the obtained compounds. The calculated results are in agreement with the experimental results.

The absorption and photoluminescence spectra were obtained and their maxima were determined. Electroluminescent investigation of pyrazoloquinoline derivatives demonstrated similar spectral features for all of the investigated compounds. Thermal measurements were also performed. Degradation temperatures in the investigated materials are high (above 300 °C). Additionally, the capillary method was used to indicate high melting temperatures. However, DSC tests showed that three out of seven (D2, D5, and D7) compounds exhibit glass transitions. Organic electroluminescent diodes were also made using the tested compounds as a dye in the PVK matrix. The fabricated diodes show various mechanisms of electroluminescence. They light up in blue-greenish colors. Taking into consideration thermal properties and ignition voltages, the best materials for application are: (4-chlorophenyl)-6-fluoro-3-methyl-1-phenyl-1H-pyrazolo[3,4-b]quinoline (F6) and 4-(4-*tert*-butylphenyl)-6-fluoro-3-methyl-1-phenyl-1H-pyrazolo[3,4-b]quinoline (F1). We arranged diodes according to the increasing ignition voltage (D5, D6, D7, and D1). As the best of the diodes, those with the lowest ignition voltage were chosen. Unfortunately, diodes D5 and D7 have glassy transitions in the temperature range required for applications, and are thus eliminated from application possibilities. Therefore, the most optimal diodes are D6 and D1.

## Figures and Tables

**Table 1:** Symbols and names of investigated compounds and substituents R1, R2, R3.

Symbol	Compound Name	R1	R2	R3
F1	4-(4-*tert*-butylphenyl)-6-fluoro-3-methyl-1-phenyl-1H-pyrazolo[3,4-b]quinoline	F	*t*-Bu	Me
		(fluorine)	(*t*-butyl)	(methyl)
F2	6-*tert*-butyl-4-(4-fluorophenyl)-3-methyl-1-phenyl-1H-pyrazolo[3,4-b]quinoline	*t*-Bu	F	Me
		(*t*-butyl)	(fluorine)	(methyl)
F3	6-ethyl-4-(4-fluorophenyl)-3-methyl-1-phenyl-1H-pyrazolo[3,4-b]quinoline	Et-	F	Me
		(ethylene)	(fluorine)	(methyl)
F4	4-(4-fluorophenyl)-3,6-dimethyl-1-phenyl-1H-pyrazolo[3,4-b]quinoline	Me	F	Me
		(methyl-CH3)	(fluorine)	(methyl)
F5	4-(4-fluorophenyl)-6-methoxy-3-methyl-1-phenyl-1H-pyrazolo[3,4-b]quinoline	OMe	F	Me
		(O-methyl)	(fluorine)	(methyl)
F6	4-(4-chlorophenyl)-6-fluoro-3-methyl-1-phenyl-1H-pyrazolo[3,4-b]quinoline	F	Cl	Me
		(fluorine)	(chlorine)	(methyl)
F7	4-(4-fluorophenyl)-3-isopropyl-6-methoxy-1-phenyl-1H-pyrazolo[3,4-b]quinoline	OMe	F	*i*-Pr
		(O-methyl)	(fluorine)	(isopropyl)

**Table 2:** Energy HOMO and LUMO levels calculated by TDDFT method by different levels of theory for investigated compounds F1–F7.

Level of Theory		B3LYP/6−31G(d,p)		B3LYP/6−31+G(d,p)		B3LYP/6−31G(d)	
Compound symbol	R1	HOMO	LUMO	HOMO	LUMO	HOMO	LUMO
F1	F (fluorine)	5.42	1.96	5.73	2.28	5.57	2.05
F2	*t*-Bu (*t*-butyl)	5.39	1.86	5.68	2.16	5.54	1.94
F3	Et (ethylene)	5.39	1.88	5.68	2.19	5.53	1.96
F4	Me (methyl)	5.39	1.88	5.69	2.19	5.54	1.95
F5	OMe (o-methyl)	5.31	1.85	5.61	2.17	5.44	1.94
F6	F (fluorine)	5.56	2.14	5.86	2.45	5.63	2.14
F7	OMe (o-methyl)	5.32	1.84	5.62	2.16	5.45	1.93

**Table 3:** Results of TDDFT calculations of λ_max_ in chloroform for the new compounds.

Compound	λ_max_ (nm)	f. Oscillator Strength
F1	416.6	0.103
	274.6	1.198
F2	405.8	3.055
	279.9	4.429
F3	408.7	0.089
	279.9	0.756
F4	406.7	0.094
	279.3	0.653
F5	415.6	0.103
	277.9	0.994
F6	420.3	0.091
	278.7	0.577
F7	413.6	0.105
	276.9	1.016

**Table 4:** Maxima of absorption and photoluminescence spectra of the investigated materials F1–F7 (λ_ex_—laser excitation wavelength).

Compounds	Absorption Maxima in Absorption Band 240–340 (nm)	Absorption Maxima in Absorption Band 400–650 (nm)	Maximum of Photoluminescence Spectra (λ_ex_ = 405 nm) (nm)	Stokes Shift (nm)
F1	275	400	490	90
F2	275	402	472	70
F3	275	410	472	62
F4	280	410	470	60
F5	282	410	480	70
F6	280	410	490	80
F7	282	410	475	65

**Table 5:** Parameters of the DSC curves.

Compound	Speed of the DSC Process (°C/min)	DSC Heating Peak(s) (°C)	DSC Cooling Peak(s) (°C)	Capillary Method m.p. (°C)	T_deg_ (°C)
F1	10	210	141, 136	218–221	340
	15	214	156		
F2	12	187	-	192–193	438
	10	175, 180	-		
F3	15	209, 216	150	161–162	350
	15	212	114		
F4	15	248	332	211–212	306
	10	240	330		
F5	10	166	104	211–212	317
	12	170	111		
F6	10	213	168	185	344
	15	207	161		
F7	10	192	-	247–249	341
	12	199	-		

**Table 6:** Electroluminescent parameters of the organic diodes D1–D7.

Organic Electroluminescent Diode	Diode Active Layer Dye + PVK Matrix	Wavelength of Electroluminescence Maximum (nm)	Ignition Voltage (V)	Current-Electroluminescence Exponent *n*
D1	F1 + PVK	487	7.3	1.8
D2	F2 + PVK	482	7.9	4.2
D3	F3 + PVK	481	8.1	3.4
D4	F4 + PVK	483	7.3	1.2
D5	F5 + PVK	482	6.2	0.8
D6	F6 + PVK	493	6.4	1.2
D7	F7 + PVK	487	7.1	2.4
